# The effect of robotic surgery on low anterior resection syndrome in patients with lower rectal cancer: a propensity score-matched analysis

**DOI:** 10.1007/s00464-024-10676-3

**Published:** 2024-02-07

**Authors:** Lei Zhang, Chenhao Hu, Jiamian Zhao, Chenxi Wu, Zhe Zhang, Ruizhe Li, Ruihan Liu, Junjun She, Feiyu Shi

**Affiliations:** 1 Department of General Surgery, The First Affiliated Hospital of Xi’an Jiaotong University, Xi’an, Shaanxi China; 2 Center for Gut Microbiome Research, Med-X Institute, The First Affiliated Hospital of Xi’an Jiaotong University, Xi’an, Shaanxi China

**Keywords:** Low anterior resection syndrome, Lower rectal cancer, Robotic surgery, Nomogram

## Abstract

**Background:**

Many patients experience anorectal dysfunction after rectal surgery, which is known as low anterior resection syndrome (LARS). Robotic systems have many technical advantages that may be suitable for functional preservation after low rectal resection. Thus, the study aimed to explore whether robotic surgery can reduce the incidence and severity of LARS.

**Methods:**

Patients undergoing minimally invasive sphincter-sparing surgery for low rectal cancer were enrolled between January 2015 and December 2020. The patients were divided into robotic or laparoscopic groups. The LARS survey was conducted at 6, 12 and 18 months postoperatively. Major LARS scores were analysed as the primary endpoint. In order to reduce confounding factors, one-to-two propensity score matches were used.

**Results:**

In total, 342 patients were enrolled in the study. At 18 months postoperatively, the incidence of LARS was 68.7% (235/342); minor LARS was identified in 112/342 patients (32.7%), and major LARS in 123/342 (36.0%). After matching, the robotic group included 74 patients, and the laparoscopic group included 148 patients. The incidence of major LARS in the robotic group was significantly lower than that in the laparoscopic group at 6, 12, and 18 months after surgery. In multivariate logistic regression analysis, tumour location, laparoscopic surgery, intersphincteric resection, neoadjuvant therapy, and anastomotic leakage were independent risk factors for major LARS after minimally invasive sphincter-sparing surgery for low rectal cancer. Furthermore, a major LARS prediction model was constructed. Results of model evaluation showed that the nomogram had good prediction accuracy and efficiency.

**Conclusions:**

Patients with low rectal cancer may benefit from robotic surgery to reduce the incidence and severity of LARS. Our nomogram could aid surgeons in setting an individualized treatment program for low rectal cancer patients.

**Supplementary Information:**

The online version contains supplementary material available at 10.1007/s00464-024-10676-3.

Multidisciplinary management of rectal cancer, including neoadjuvant therapy, total mesorectal excision (TME), and advanced surgical techniques is not only beneficial for reducing local recurrence but also can increase the rate of sphincter-preserving rectal resection [1–3]. However, postoperative rectum dysfunction, also reported as low anterior resection syndrome (LARS), has been exacerbated by optimized sphincter-preserving rates. In a growing number of studies, LARS prevalence has ranged from 16.0 to 57.9% for major LARS [4–6].

The exact cause of LARS is unclear, but it appears that surgery may damage the anatomical structure and physiological functions of normal defecation [7, 8]. Additionally, several representative factors have been reported to be implicated with LARS, including a lesion of the anal sphincter, the location of anastomosis, neoadjuvant radiotherapy, the presence of defunctioning ileostomy and anastomotic leakage (AL) [9, 10]. Lower rectal tumours are located in the deep and narrow pelvis; consequently, it is extremely technically challenging to dissociate perirectal structures. Thus, the lower the tumour location is, the more frequently LARS occurs. Many studies have shown that robot-assisted surgery is associated with better short-term outcomes and even better preservation of urogenital function in patients with low rectal cancer [11, 12]. Thus, robotic surgery may serve as a potential preventive approach for LARS. However, as far as we know, no direct prospective comparison of postoperative anorectal function between robotic surgery and laparoscopic surgery exists.

Thus, a prospective study of minimally invasive sphincter-saving surgeries was conducted to evaluate the relationship between the type of surgery, perioperative variables and the incidence and severity of LARS.

## Materials and methods

### Study population

Patients who underwent minimally invasive low anterior resection (LAR) or intersphincteric resection (ISR) for rectal cancer between January 2015 and December 2020 were screened for inclusion. Patient exclusion criteria included (a) tumours with a distal margin above the peritoneal reflection; (b) who had not answered the LARS questionnaire; (c) who experienced local recurrence and distant metastasis at the postoperative follow-up; (d) who had a permanent stoma; and (e) who were lost to follow-up. The Ethics and Human Subject Committee of the First Affiliated Hospital of Xi'an Jiaotong University approved the study protocol (2019-ZD-04). All study participants provided comprehensive written informed consent.

### LARS survey and data collection

LARS is diagnosed by assessing the LARS scale scores [13]. All patients are classified into 3 groups according to their questionnaire score: absence of LARS (scores of 0–20), minor LARS (21–29), and major LARS (30 to 42). The LARS score was calculated from postoperative follow-up surgical visit reports or from telephone interviews after ensuring informed consent was obtained. At fixed postoperative time points (6, 12, and 18 months), patients were asked to complete the questionnaire by themselves with or without family support. The statistical analysis was based on the postoperative 18-month survey data.

Patient demographics and perioperative data were collected from the prospective rectal cancer database of our institution. At our institution, the choice between laparoscopic and robotic operation was based on the preoperative imaging evaluation, general condition of patients, the experience of the surgon, and willingness of the patients. ISR was recommended for the patients with distal rectal tumors without infiltration of the external sphincter. For detailed surgical procedures are given in Supplementary Materials. The location of the tumour and whether the lower margin of the tumour was located below the peritoneal reflection were evaluated by preoperative imaging examination. The 8th edition of AJCC classification system was used to define the cancer stage [14]. AL was determined by retrospective analysis of medical and imaging records [15].

### Propensity score matching (PSM)

PSM was used to adjust for potential confounding factors. Thus, age, sex, body mass index (BMI), tumour distance from the anal verge, American Society of Anesthesiologists (ASA) score, previous history of abdominal surgery, operative method, construction of diverting ileostomy, lateral lymph node dissection, pathological stage, neoadjuvant treatment, and adjuvant treatment were identified as match parameters. The matching on the propensity score (1:2) was performed using a nearest neighbour-matching algorithm, with a maximum calliper distance of 0.2 of the standard deviation of the logit of the propensity score.

### Statistical analysis

Statistical analysis was performed using SPSS (Version 26.0; IBM, New York, NY, *USA*) and R(Version 4.1; *R* Foundation, Vienna, Austria). All categorical data are presented as the number of cases and percentages, while continuous data are shown as the median and interquartile range (IQR). Student’s *t* test or the Mann‒Whitney *U* test was used for continuous variables, and for categorical variables, the *χ*^*2*^ test was used. Univariate and multivariate logistic regression analyses were performed to identify independent risk factors associated with major LARS. The nomogram was built based on the results of multivariate analyses. Receiver operating characteristic curves, calibration curves and decision curve analysis curves were used to evaluate the efficiency of the prediction model.

## Results

**Fig. 1:**
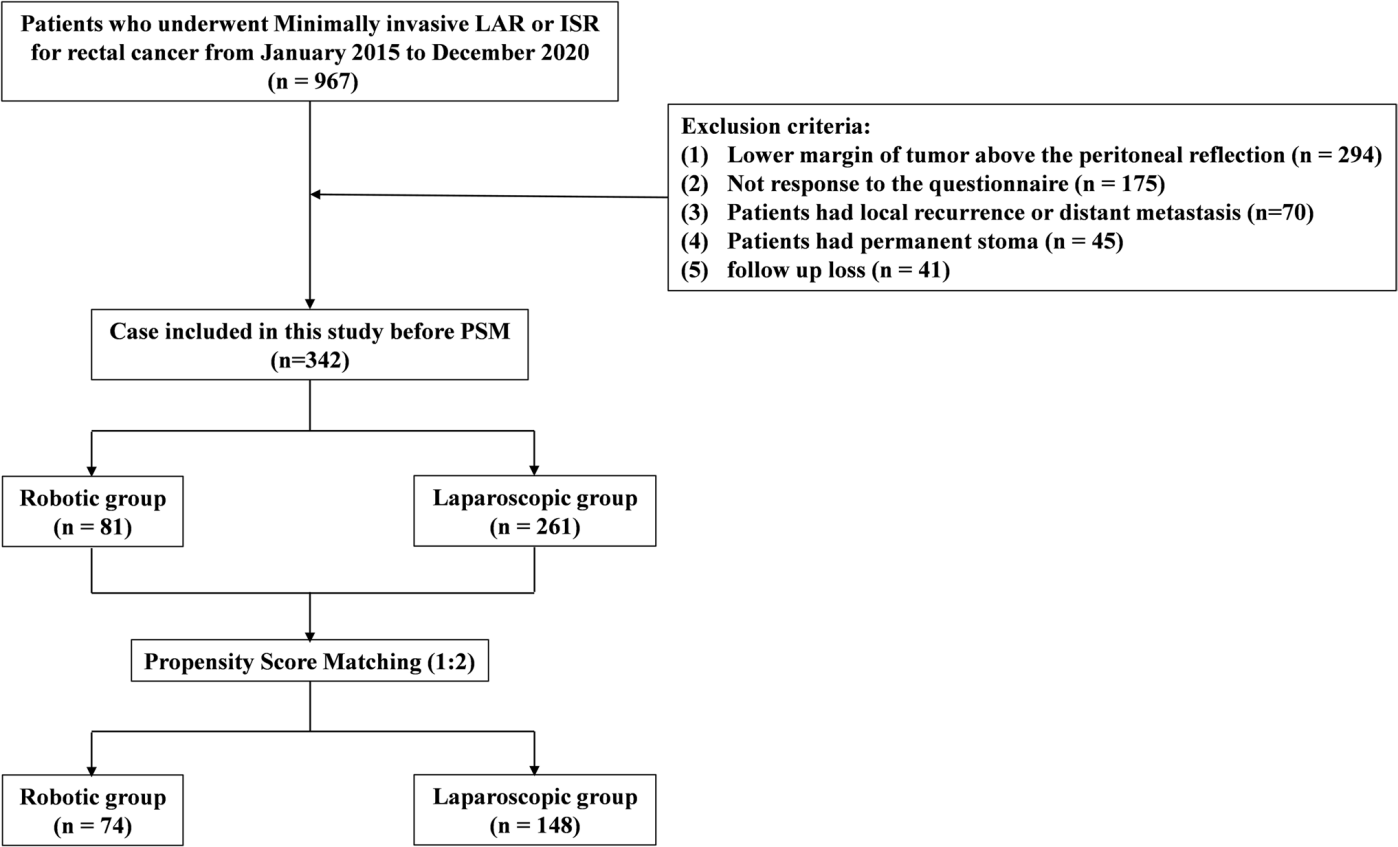
Flow chart of study patients.* LAR* low anterior resection,* ISR* intersphincteric resection, *PSM* propensity score matching

Between January 2015 and December 2020, 967 rectal cancer patients underwent LAR or ISR at our institution. After exclusions, a total of 342 patients were enrolled in the study. Among the 342 patients, 81 (23.7%) underwent robotic surgery, and 261 (76.3%) underwent laparoscopic surgery. After 1:2 matching, 74 patients who underwent robotic surgery and 148 patients who underwent laparoscopic surgery were eventually included in the analysis (Fig. 1).

The baseline characteristics before and after matching are presented in Table 1. Before PSM, the distribution of the ASA score and the proportion of lateral lymph node dissection were significantly different between the two groups (all *P* < 0.05). After PSM, there were no statistically differences between the groups for the matched covariates. Before and after PSM, the LARS score was significantly different between the robotic group and laparoscopic group (20.14 ± 13.02 vs. 24.93 ± 11.45, *P* = 0.039; 20.10 ± 13.05 vs. 25.28 ± 10.23, *P* = 0.001). Moreover, the proportion of major LARS was also significantly lower in the robotic group than in the laparoscopic group (25.9% vs. 39.1%, *P* = 0.019; 24.3% vs. 39.2%, *P* = 0.005).

**Table 1:** Baseline characteristics of enrolled patients before and after propensity score matching

Variable		Before matching		*p*-value	After matching		*p*-value
		Robotic group (*n* = 81)	Laparoscopic group (*n* = 261)		Robotic group (*n* = 74)	Laparoscopic group (*n* = 148)	
Age, n (%)				0.065			0.216
≤ 70		48 (59.3)	124 (47.5)		44 (59.3)	75 (50.7)	
> 70		33 (41.7)	137 (52.5)		30 (41.7)	73 (49.3)	
Sex, n (%)				0.176			0.376
Male		54 (66.7)	152 (58.2)		49 (66.2)	89 (60.1)	
Female		27 (33.3)	109 (41.8)		25 (33.8)	59 (39.9)	
ASA score, n (%)				0.010			0.233
I, II		58 (66.7)	145 (58.2)		52 (70.3)	92 (62.2)	
III, IV		23 (33.3)	116 (41.8)		22 (29.7)	56 (37.8)	
BMI, kg/m^2^				0.752			0.917
≤ 25		54 (66.7)	169 (64.8)		53 (71.6)	82 (70.9)	
> 25		27 (33.3)	92 (35.2)		21 (28.4)	66 (29.1)	
Distance to the anal verge, cm		5.1 ± 1.3	5.8 ± 1.8	0.276	5.1 ± 1.2	5.3 ± 1.5	0.624
Previous abdominal surgery, n (%)		11 (13.6)	39 (14.9)	0.762	11 (14.9)	23 (15.5)	0.895
Type of surgery, n (%)				0.506			0.621
ISR		12 (14.8)	47 (18.0)		12 (16.2)	28 (18.9)	
LAR		69 (85.2)	214 (72.0)		62 (83.8)	120 (81.1)	
Construction of diverting ileostomy, n (%)		46 (56.8)	167 (64.0)	0.243	43 (56.8)	91 (64.0)	0.628
Lateral lymph node dissection n (%)		21 (25.9)	36 (13.8)	0.010	19 (25.7)	26 (17.6)	0.157
Pathological TNM stage, n (%)				0.719			0.959
I		13 (16.0)	34 (14.2)		12 (16.2)	25 (16.9)	
II		37 (45.7)	113 (42.5)		32 (43.2)	61 (41.2)	
III		31 (38.3)	114 (43.3)		30 (40.5)	62 (41.9)	
Neoadjuvant therapy, n (%)		39 (48.1)	113 (43.3)	0.443	36 (48.6)	67 (46.6)	0.776
Adjuvant chemotherapy, n (%)		49 (60.5)	142 (54.4)	0.335	44 (59.5)	76 (51.4)	0.253
LARS score		20.14 ± 13.02	24.93 ± 11.45	0.039	20.10 ± 13.05	25.28 ± 10.23	0.001
LARS classification, n (%)				0.019			0.005
No		35 (43.2)	72 (27.6)		33 (44.6)	35 (23.6)	
Minor		25 (30.9)	87 (33.3)		23 (31.1)	55 (37.2)	
Major		21 (25.9)	102 (39.1)		18 (24.3)	58 (39.2)	

*LARS* low anterior resection syndrome, *ASA* American society of Aneshesiologists, *BMI* body mass index, *LAR* low anterior resection, *ISR* intersphincteric resection, *TNM* Tumor Node Metastasis

The frequency of the individual components of LARS is summarized in Table 2. In addition to flatus incontinence, there were significant differences between the robotic and laparoscopic groups in liquid stool status ([at least once a week] 17.6% vs. 33.8%, *P* = 0.020), frequency ([more than 7 times per day] 5.4% vs. 21.6%, *P* < 0.001), clustering ([at least once a week] 43.2% vs. 39.2%, *P* = 0.010), and urgency ([at least once a week] 21.6% vs. 39.2%, *P* = 0.018).

**Table 2:** Incidence of LARS and score components

Variable		Robotic group (*n* = 74)	Laparoscopic group (*n* = 148)	*p*-value
LARS classification, n (%)				0.005
No		33 (44.6)	35 (23.6)	
Minor		23 (31.1)	55 (37.2)	
Major		18 (24.3)	58 (39.2)	
Cannot control flatus				0.664
No, never		38 (51.4)	83 (56.1)	
Yes, less than once per week		28 (37.8)	47 (31.8)	
Yes, at least once per week		8 (10.8)	18 (12.2)	
Liquid stool leakage				0.020
No, never		57 (77.0)	86 (58.1)	
Yes, less than once per week		4 (5.4)	12 (8.1)	
Yes, at least once per week		13 (17.6)	50 (33.8)	
How often open bowels				< 0.001
Less than once per day (24 h)		9 (12.2)	40 (27.1)	
1–3 times per day (24 h)		42 (56.8)	44 (29.7)	
4–7 times per day (24 h)		19 (25.7)	32 (21.6)	
More than 7 times per day (24 h)		4 (5.4)	32 (21.6)	
Open bowels within 1 h				0.010
No, never		22 (29.8)	23 (15.5)	
Yes, less than once per week		20 (27.0)	67 (45.3)	
Yes, at least once per week		32 (43.2)	58 (39.2)	
Strong urge to open bowels				0.018
No, never		23 (31.1)	28 (18.9)	
Yes, less than once per week		35 (47.3)	62 (41.9)	
Yes, at least once per week		16 (21.6)	58 (39.2)	

*LARS* low anterior resection syndrome

**Fig. 2:**
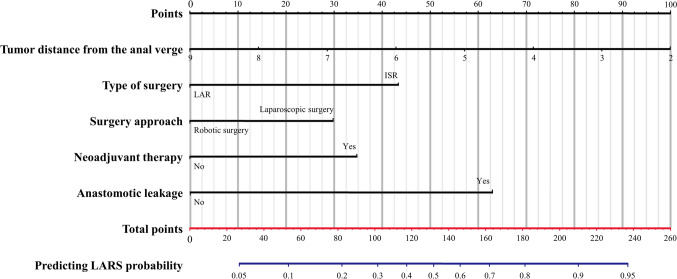
Nomogram of major LARS in patients with low rectal cancer. *LAR* low anterior resection, *ISR* intersphincteric resection, *LARS*, low anterior resection syndrome

Table 3 provides the univariate analysis and the potential risk factors. In univariate analysis, we found that major LARS was significantly associated with the ASA score, tumour location, type of surgery, surgical approach, diverting ileostomy, neoadjuvant therapy, and AL. Multivariate logistic analysis further showed that tumour location [odds ratio (OR): 0.676, 95% confidence interval (CI) 0.561–0.814, *P* < 0.001], ISR (OR: 3.297, 95% CI 1.468–7.399, *P* = 0.004), laparoscopic surgery (OR: 2.244, 95% CI 1.064–4.732, *P* = 0.034), neoadjuvant therapy (OR: 2.541, 95% CI 1.303–4.954, *P* = 0.006) and AL (OR: 5.102, 95% CI 2.075–12.547, *P* < 0.001) were independent predictors of major LARS (Table 4). Based on the independent predictive variables, we constructed a predictive model for major LARS (Fig. 2). The area under the receiver operating characteristic curve of the nomogram for predicting major LARS was 0.812 (95% CI 0.752–0.871) (Supplementary Fig. 1). The calibration curves suggested that the predicted calibration curve was nearly coincident with the standard curve, which indicated that the nomogram performed well in predicting major LARS (Supplementary Fig. 2**)**. Moreover, the decision curve analysis showed that the model had good clinical application efficacy for predicting major LARS (Supplementary Fig. 3).

**Table 3:** Distribution and analysis of possible LARS risk factors

Risk factor		*n*(%)	LARS			*p*-value
			No LARS (*n* = 68)	Minor LARS (*n* = 78)	Major LARS (*n* = 76)	
Age						0.408
≤ 70		119 (53.6)	41 (34.5)	39 (32.8)	39 (32.8)	
> 70		103 (46.4)	27 (26.2)	39 (37.9)	37 (35.9)	
Sex						0.056
Male		138 (62.2)	49 (35.5)	49 (35.5)	40 (29.0)	
Female		84 (37.8)	19 (22.6)	29 (34.5)	36 (42.9)	
ASA score						0.022
I, II		144 (64.9)	48 (33.3)	56 (38.9)	40 (27.8)	
III, IV		78 (35.1)	20 (25.6)	22 (28.2)	36 (46.2)	
BMI, kg/m^2^						0.606
≤ 25		135 (60.8)	38 (28.1)	49 (36.3)	48 (35.6)	
> 25		87 (39.2)	30 (34.5)	29 (33.3)	28 (32.2)	
Distance to the anal verge, mean ± SD, cm		–	6.6 ± 0.7	5.2 ± 0.6	4.3 ± 0.7	< 0.001
Previous surgery						0.278
Yes		34 (15.3)	9 (26.5)	16 (47.1)	9 (26.5)	
No		188 (84.7)	59 (31.4)	62 (33.0)	67 (35.6)	
Type of surgery						0.008
ISR		40 (18.0)	7 (17.5)	11 (27.5)	22 (55.0)	
LAR		182 (82.0)	61 (33.5)	67 (36.8)	54 (29.7)	
Surgery approach						0.005
Robotic surgery		74 (33.3)	33 (44.6)	23 (31.1)	18 (24.3)	
Laparoscopic surgery		148 (66.7)	35 (23.6)	55 (37.2)	58 (39.2)	
Construction of diverting ileostomy						0.004
Yes		134 (60.4)	30 (22.4)	51 (38.1)	53 (39.6)	
No		88 (39.6)	38 (43.2)	27 (30.7)	23 (26.1)	
Time of ileostomy closure						0.460
Early (< 3 months)		42 (31.3)	16 (38.1)	12 (28.6)	14 (33.3)	
Late (> 3 months)		92 (68.7)	36 (39.1)	34 (37.0)	22 (23.9)	
Lateral lymph node dissection						0.096
Yes		45 (20.3)	9 (20.0)	15 (33.3)	21 (46.7)	
No		177 (79.7)	59 (33.3)	63 (35.6)	55 (31.1)	
T stage						0.494
T1-2		57 (25.7)	14 (24.6)	21 (36.8)	22 (38.6)	
T3-4		165 (74.3)	54 (32.7)	57 (34.5)	54 (32.7)	
N stage						0.263
N0		130 (58.6)	36 (27.7)	44 (33.8)	50 (38.5)	
N1-2		92 (41.4)	32 (34.8)	34 (37.0)	26 (28.3)	
Pathological TNM stage						0.519
I		37 (16.7)	9 (24.3)	12 (32.4)	16 (43.2)	
II		93 (41.9)	27 (29.0)	32 (34.4)	34 (36.6)	
III		92 (41.4)	32 (34.8)	34 (37.0)	26 (28.2)	
Neoadjuvant therapy						< 0.001
Yes		103 (46.4)	23 (22.3)	31 (30.1)	49 (47.6)	
No		119 (53.6)	45 (37.8)	47 (39.5)	27 (22.7)	
Adjuvant chemotherapy						0.768
Yes		120 (54.1)	39 (32.5)	42 (35.0)	39 (32.5)	
No		102 (45.9)	29 (28.4)	36 (35.3)	37 (36.3)	
Operation time, median (IQR), min		–	205 (66)	200 (82)	200 (74)	0.317
Blood loss, median (IQR), ml		–	100 (75)	75 (100)	100 (100)	0.645
Anastomotic leakage						< 0.001
Yes		36 (16.2)	2 (5.6)	11 (30.6)	23 (63.9)	
No		186 (83.8)	66 (35.5)	67 (36.0)	53 (28.5)	

*LARS* low anterior resection syndrome, *ASA* American society of Aneshesiologists, *BMI* body mass index, *LAR* low anterior resection, *ISR* intersphincteric resection, *TNM* Tumor Node Metastasis,* IQR* interquartile range

**Table 4:** Univariate and multivariate analysis of clinical characteristics associated with major LARS

Variables	Univariate analysis		Multivariate analysis	
	Odds ratio (95% CI)	***p***-value	Odds ratio (95% CI)	***p***-value
ASA III or IV (vs ASA I or II)	2.086 (1.177–3.698)	0.012	1.383 (0.698–2.714)	0.353
Tumor location (1 cm increase)	0.686 (0.584–0.807)	< 0.001	0.676 (0.561–0.814)	< 0.001
ISR (vs LAR)	2.897 (1.440–5.831)	0.003	3.297 (1.468–7.399)	0.004
Laparoscopic surgery (vs robotic surgery)	2.005 (1.073–3.747)	0.029	2.244 (1.064–4.732)	0.034
Diverting ileostomy (vs no diverting ileostomy)	1.849 (1.027–3.330)	0.041	1.426 (0.716–2.839)	0.313
Neoadjuvant therapy (vs no neoadjuvant therapy)	3.092 (1.735–5.509)	< 0.001	2.541 (1.303–4.954)	0.006
Anastomotic leakage (vs no anastomotic leakage)	4.440 (2.095–9.048)	< 0.001	5.102 (2.075–12.547)	< 0.001

*ASA* American society of Aneshesiologists, *LAR* low anterior resection, *ISR* intersphincteric resection

**Fig. 3:**
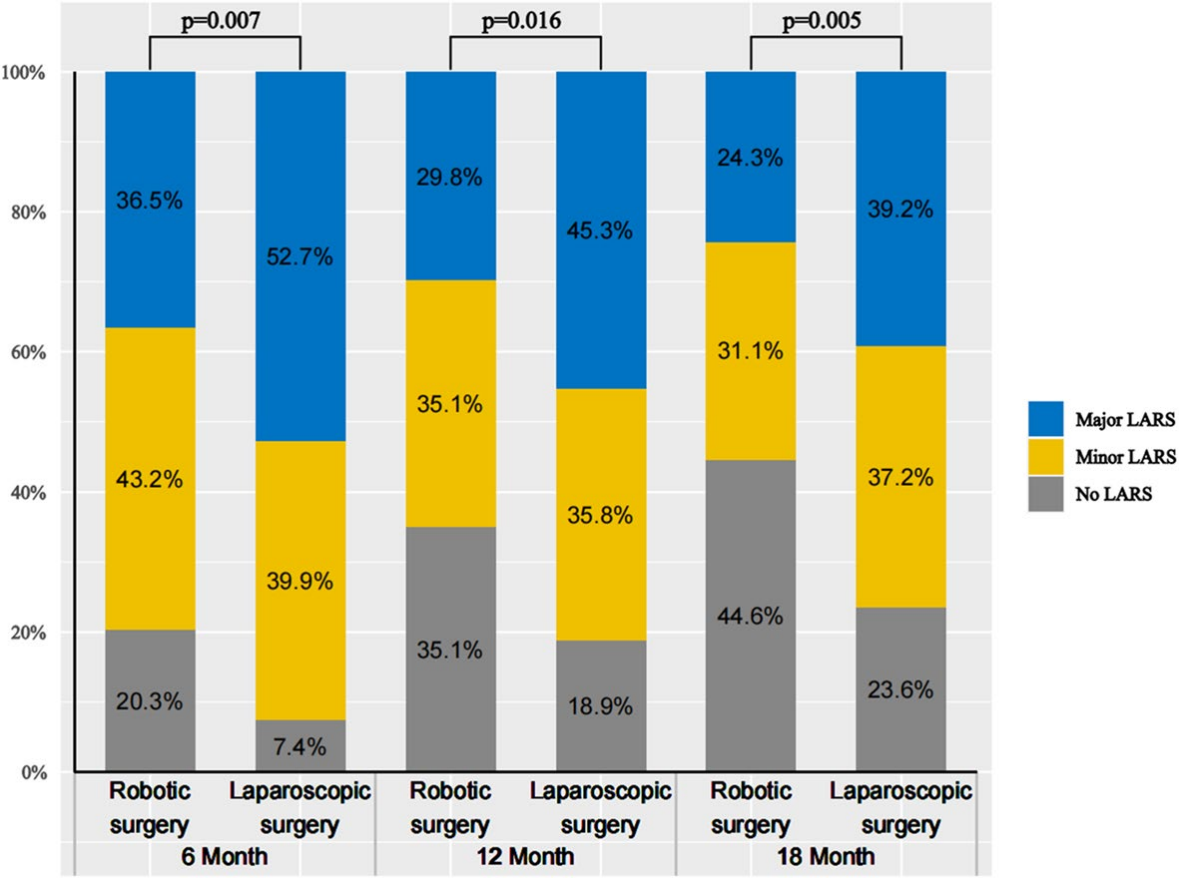
Variations of LARS score of robotic and laparoscopic groups over time. *LARS* low anterior resection syndrome

Figure 3 shows the changes in LARS scores at different follow-up time points after surgery. The greatest occurrence of “major” LARS was observed at 6 months [36.5% (robotic group) vs. 52.7% (laparoscopic group); *P* = 0.007]; it was diminished at 12 months [29.8% (robotic group) vs. 45.3% (laparoscopic group); *P* = 0.016] and, further, at 18 months [24.3% (robotic group) vs. 39.2% (laparoscopic group); *P* = 0.005]. With follow-up progression, common LARS severity subgroups emerged, namely, subgroups representing a change from “major” towards “minor” LARS and from “minor” to “no” LARS. Statistically significant differences between groups according to the LARS score incidence persisted from 6 months postoperatively to 18 months postoperatively.

## Discussion

Due to the issues of oncological results and functional restoration, low rectal cancers may present a difficult technical challenge even to experienced colorectal surgeons. Although laparoscopic surgical techniques have been widely applied to lower rectal resection, there are still many controversies about functional outcomes [16, 17]. Robotic platforms offer obvious advantages such as 3D visualization, flexible robotic arms, and precise movements, which may be more beneficial to the retention of functionality [18]. Our study is the first prospectively and dynamically analyse the association between LARS and robotic surgery. Our results showed that robotic surgery significantly reduced the incidence and severity of LARS in low rectal cancer patients compared with laparoscopic surgery.

Surgical advances, precise instrumentation and surgical staplers have significantly improved the sphincter-preserving rate of low rectal cancer. However, the maintained sphincter function, reduced neorectum and maximum tolerated volume, and increased frequency and urgency of bowel movements lead to the occurrence of LARS. The exact diagnosis of LARS depends on adequate evaluation of the patient’s symptoms. However, the impact of LARS is heterogeneous and influenced by personalities and emotions, and a limited and uneven follow-up period may not accurately measure the extent of the problem. According to a meta-analysis, in more than 70% of studies, no further verification of the LARS questionnaire was used [19]. A recent systematic review also stated that LARS symptoms are time dependent, generally regressing in a variable interval from 6 to 18 months, which is the time necessary for the neo-rectum to “rehabilitate” and beyond which further improvement is unlikely [20]. Therefore, this study selected 6 months, 12 months and 18 months after surgery as fixed follow-up times for the LARS survey. The follow-up data 18 months after the operation were used as our primary endpoint for outcome analyses.

Based on multivariate analysis, we identified tumour location, laparoscopic surgery, ISR, neoadjuvant therapy, and AL as independent risk factors for major LARS. Ekkarat et al*.* showed that tumour located < 5 cm from the anal verge was a risk factor for severe LARS [21]. Our results demonstrated that as the lower margin of the tumour approached the anal verge, the incidence of LARS increased. This could be due to several reasons. First, a major challenge in low rectal cancer surgery is the poor visualization of the deep pelvis, which makes it difficult to reach the target anatomy and surgical plane. Thus, LARS may be caused by physiological changes following damage to the perirectal structures and their innervation. Next, the incidence and severity of LARS is also affected by the length of the residual rectum, and it has been reported that the shorter the residual rectum is, the higher the incidence and severity of LARS [22]. Therefore, full attention should be given to the problem of LARS after surgery for patients with low tumour location.

Regarding the impact of robotic surgery on postoperative rectum defecation, there are few relevant data and somewhat clinical heterogeneity. In Masakatsu’s study, no evident preponderance of robotic surgery in preventing LARS was observed [23]. However, the ROLARR trial demonstrated that robotic platforms can be beneficial for sphincter-preserving operations [18]. In our study, we demonstrated that robotic surgery is a protective factor against major LARS (*OR*: 2.244, 95% *CI* 1.064–4.732,* P* = 0.034). The result may be due to the fact that our study enrolled patients with low rectal cancer, which is related to the difficulty of surgery. The limited dexterity of laparoscopic instruments leads to difficulties during low rectal cancer surgery. However, meticulous dissection in critical steps is a potential technical advantage of robotic systems. The three-dimensional field of vision and flexible manipulator arm in low rectal dissection allow better visualization of the anatomical planes. Moreover, eliminating tremors and improving the accuracy of cutting by robotic systems allow better exposure and protection of the pelvic autonomic nerve during TME, which probably results in less anorectal dysfunction.

Neoadjuvant therapy has become the standard treatment for advanced low rectal cancer [24]. Our study showed that neoadjuvant therapy was significantly associated with an increased probability of major LARS. It is well-known that radiotherapy produces biological alterations in cancer cells [25]. Neoadjuvant radiotherapy induces neural damage, ischaemic changes in the intestinal mucosa and sphincter fibrosis. Because of this, the neorectum is sensitive to mechanical stimulation, and a small amount of stool can cause it to contract. In Antonella’s study, patients undergoing neoadjuvant therapy had a more severe form of LARS (*OR*: 2.18, 95% *CI* 1.00–4.78, *P* = 0.05) [26], which is similar to our results.

ISR is a safe anus-preserving operation for patients with ultra-low rectal cancer. This technique makes it easier to avoid extremely low anatomy and difficult abdominal stapling. Thus, ISR is particularly suitable for patients with excessive obesity, narrow pelvis and tumour located < 5 cm over the anal sphincter. However, the approach has raised concerns about the potential impact on postoperative anorectal functions due to mechanical resection of part of the internal anal sphincter, which is responsible for approximately 70% of anal static tension [27]. Gori et al. [28] also reported that the incidence of major LARS was significantly higher in ISR group than in LAR group (25% versus 11%). Thus, transabdominal stapling may be pursued until tumour volume or adequate distal margin is not feasible, when conventional ISR should be performed.

AL is the most severe surgical complication in rectal surgery and is considered a risk factor for recovery of rectum function. AL causes inflammatory changes in the pelvis, which may cause pelvic autonomic nerve lesions. Furthermore, rectal healing after AL can be associated with excessive fibrotic scarring, which could eventually alter compliance of the neorectum. Several studies have shown that AL increases the risk of major LARS [29, 30]. Thus, targeted prevention measures should be formulated to minimize the incidence of postoperative LARS and improve the quality of life of rectal cancer patients.

In these circumstances, the risk of LARS and its impact on quality of life must be taken into appropriate consideration in the management of low rectal cancer. It is the responsibility of surgons to explain the benefits of different treatment options and warn of the potential risks of long-term anorectal dysfunction. Our predictive model allowed us to quantify the risk of severe LARS, which is important for patient-clinician decision making. Only when patients have a full understanding of LARS will they be more active in adopting biofeedback, sacral nerve regulation, rectal irrigation and other treatments to improve their postoperative anorectal function and quality of life.

There were several limitations to this study. First, it had a nonrandomized study design. Patients were categorized into robotic or laparoscopic groups according to the type of surgery, which may create some degree of misclassification bias. Due to the technical advantages of robotic surgery, patients with ultra-low rectal cancer or large rectal tumor may be more likely to receive robotic surgery. In addition, patients with male, obesity, prostatic hypertrophy and narrow pelvis also tend to choose robotic surgery. Thus, we performed PSM to minimize selection bias. Next, this study selected 6 months, 12 months and 18 months after surgery as fixed follow-up times for LARS. A small number of studies have reported LARS results at 3 years and even 5 years postoperatively using retrospective analysis. We also followed up with some patients 2 years after surgery and found no significant changes compared to 18 months after surgery. Thus, we did not conduct further follow-up. We look forward to further prospective, multicentre, repetitive, longitudinal, and large-sample randomized control trials to confirm our findings.

## Conclusion

Robotic surgery may help to reduce the incidence and severity of LARS in patients with low rectal cancer. Tumour location, ISR, neoadjuvant therapy, and AL were also independent risk factors for severe LARS, which should be considered when formulating individualized treatment strategies.

### Supplementary Information

Below is the link to the electronic supplementary material.

Supplementary file1 (DOCX 385 KB)

## Data Availability

For more information on raw data and analysis methods or research materials, please contact the corresponding author.
