# Supplementary material for: The effect of robotic surgery on low anterior resection syndrome in patients with lower rectal cancer: a propensity score-matched analysis
Source: Surg Endosc. 2024 Feb 7;38(4):1912–21. doi: 10.1007/s00464-024-10676-3 (PMC10978601; doi:10.1007/s00464-024-10676-3)
Supplement: Supplementary file 1 — Supplementary file1 (DOCX 385 KB) [file 464_2024_10676_MOESM1_ESM.docx]

**The effect of robotic surgery on** **low anterior resection syndrome in patients with lower rectal cancer: A propensity score-matched analysis**

Zhang et al.

(Supplementary materials)

**Supplementary Methods**

**Operative technique**

Low anterior resection (LAR) was performed in patients whose tumour was ≥4cm from the anal verge. Intersphincteric resection (ISR) was considered for the patients with distal rectal tumours without infiltration of the external sphincter. Patients with T4 tumours, poorly differentiated tumours and no response to neoadjuvant therapy were contraindicated for ISR. The choice between laparoscopic and robotic operation was based on the preoperative imaging evaluation, general condition of patients, the experience of the surgon, and willingness of the patients. The sequence of surgical steps for both the robotic and laparoscopic procedures was identical, except for the port position. Detailed surgical procedures were performed as follows.

LAR

After the pneumoperitoneum was established, the liver and the entire abdomen were first carefully examined for evidence of carcinogenesis. Then the trunk of the inferior mesenteric artery was separated along the root of the sigmoid colon. A low ligation of the inferior mesenteric artery and vein was performed, and the 253# lymph nodes were completely swept. Next, along the Toldts gap, the bowel was separated to sigmoid colon and descending colon junction where, according to the total mesorectal excision principle to isolate intestine to down edge of tumour and intraluminal stapler was used for mutilation of intestinal tube. Ensure that the rectum is transected below the distal border of the tumor with a distal margin of 2 cm or greater whenever possible.The hypogastric nerve plexus and pelvic plexuses should be preserved during the procedure. If lateral lymph node metastasis was suspected on preoperative evaluation, lateral lymph node dissection was further performed. At last, stopped pneumoperitoneum, taken out the separated intestinal tube from abdominal cavity by making a 4 cm oblique incision at the left lower abdomen and cut the intestinal tube at the safe distance from tumour. Pouch suturing intestinal tube and embedding anastomat were made and pneumoperitoneum was re-established. According to the situation of operation, ileostomy were made.

ISR

The initial surgical procedure was the same as described above for LAR. During rectum mobilization, the dissection was performed as low as possible to the pelvic floor towards the puborectalis and the internal sphincter to facilitate the perineal dissection. Next, the perineal transanal procedure was performed, and circumferential dissection of the intersphincteric groove was accomplished while obtaining optimal distal margin of the tumour and mucosa of the internal sphincter. The extent of internal sphincter resection determined whether a partial or total ISR was needed. The distal resection margin was reached above the intersphincteric groove in partial ISR, and the margin obtained in total ISR included the intersphincteric groove. After freely handling the rectal stump with removal of the resected rectum through the anus, a pull-through procedure (coloanal anastomosis) was performed with hand-sewn interrupted absorbable sutures. At last, covering loop ileostomy was routinely employed.

Free dissection of pelvic visceral fascia, internal iliac vascular sheath and paravascular lymph nodes, lymphatic tissue in the bladder cavity, and routine pathological examination of excised specimens were carried out after surgery.

**Supplementary Results**

**
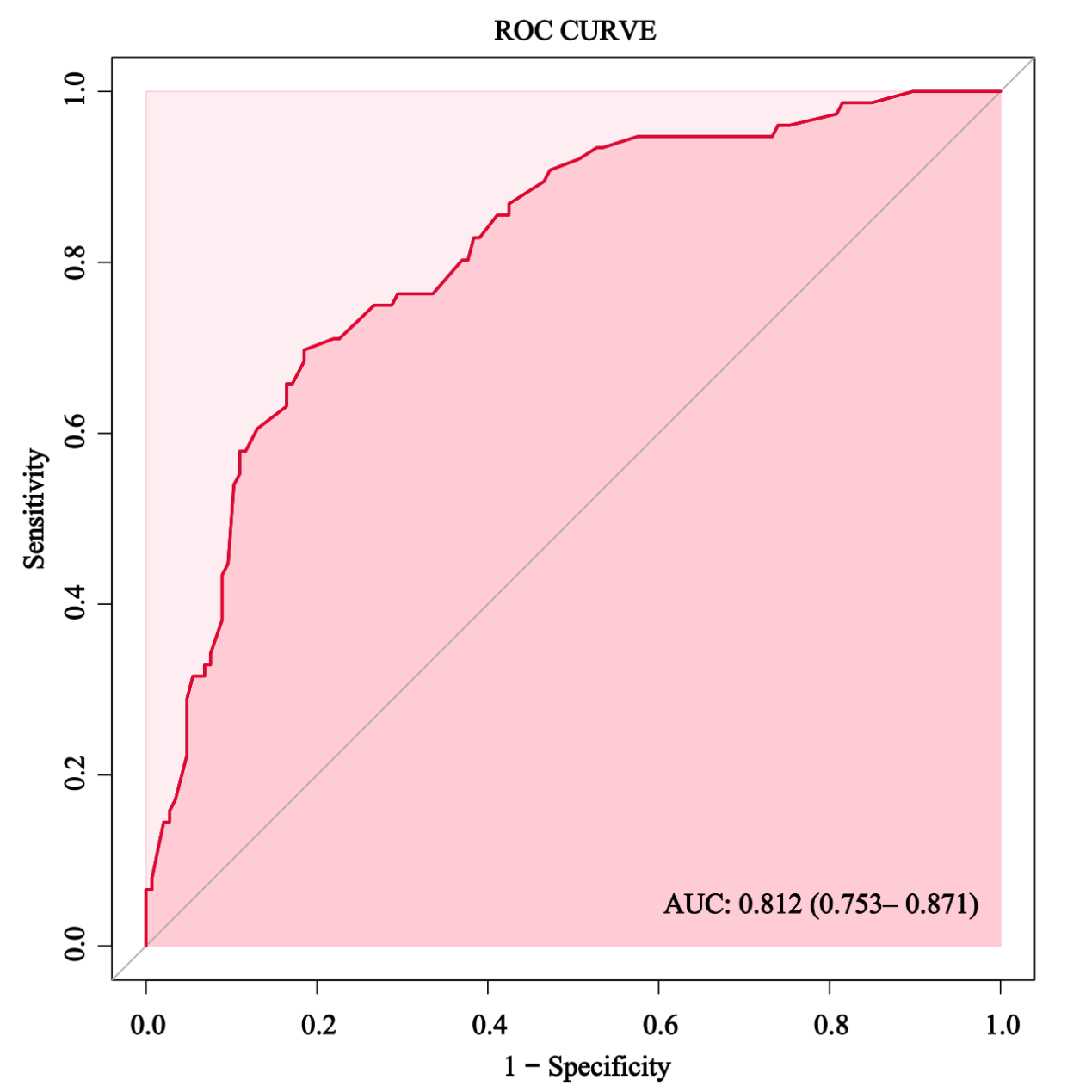
**

**Supplementary Fig. 1** The ROC curve of the nomogram for predicting major LARS. ROC, receiver operator characteristic.

**
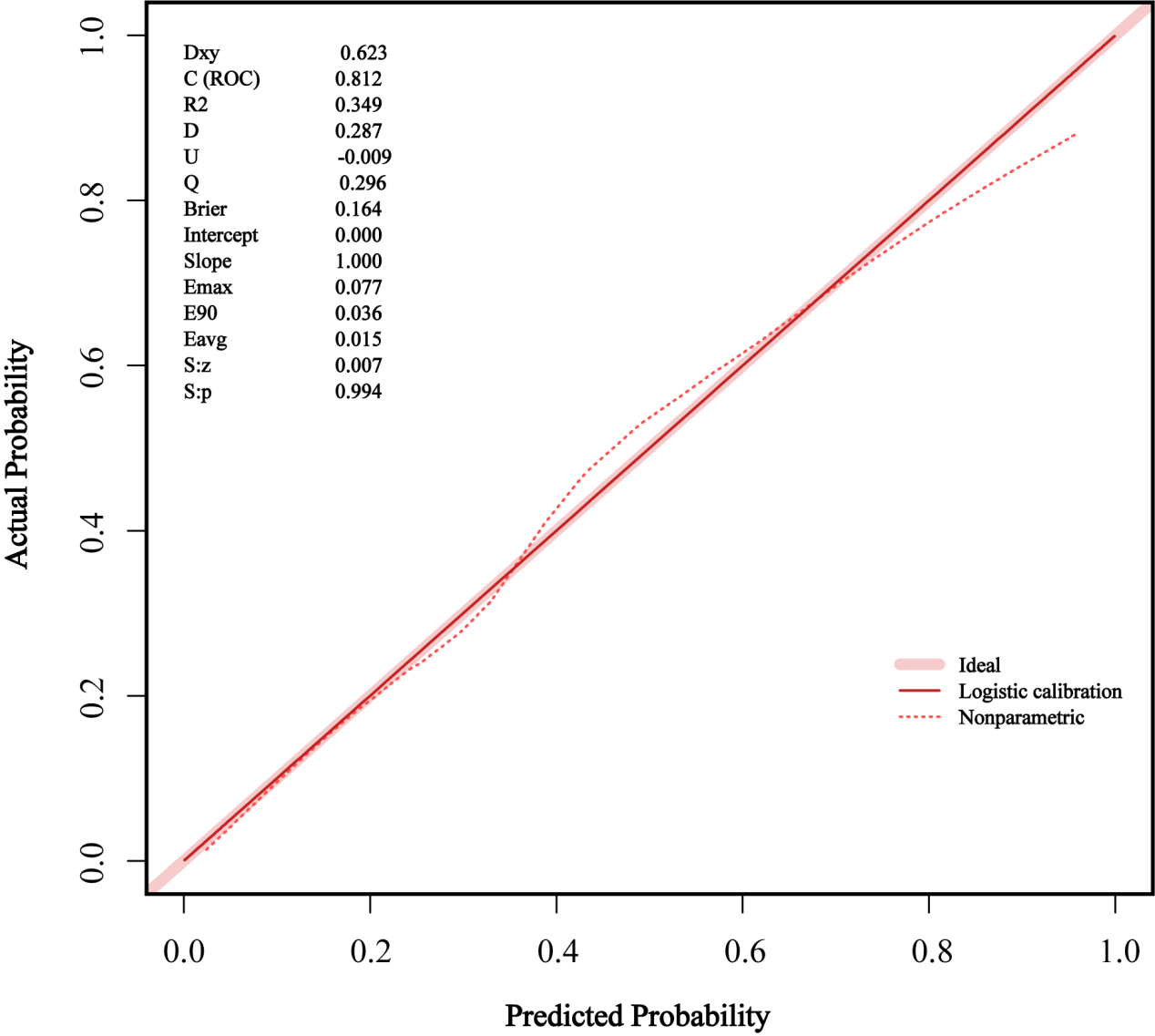
**

**Supplementary Fig. 2** The calibration curves of the nomogram for predicting major LARS.

**
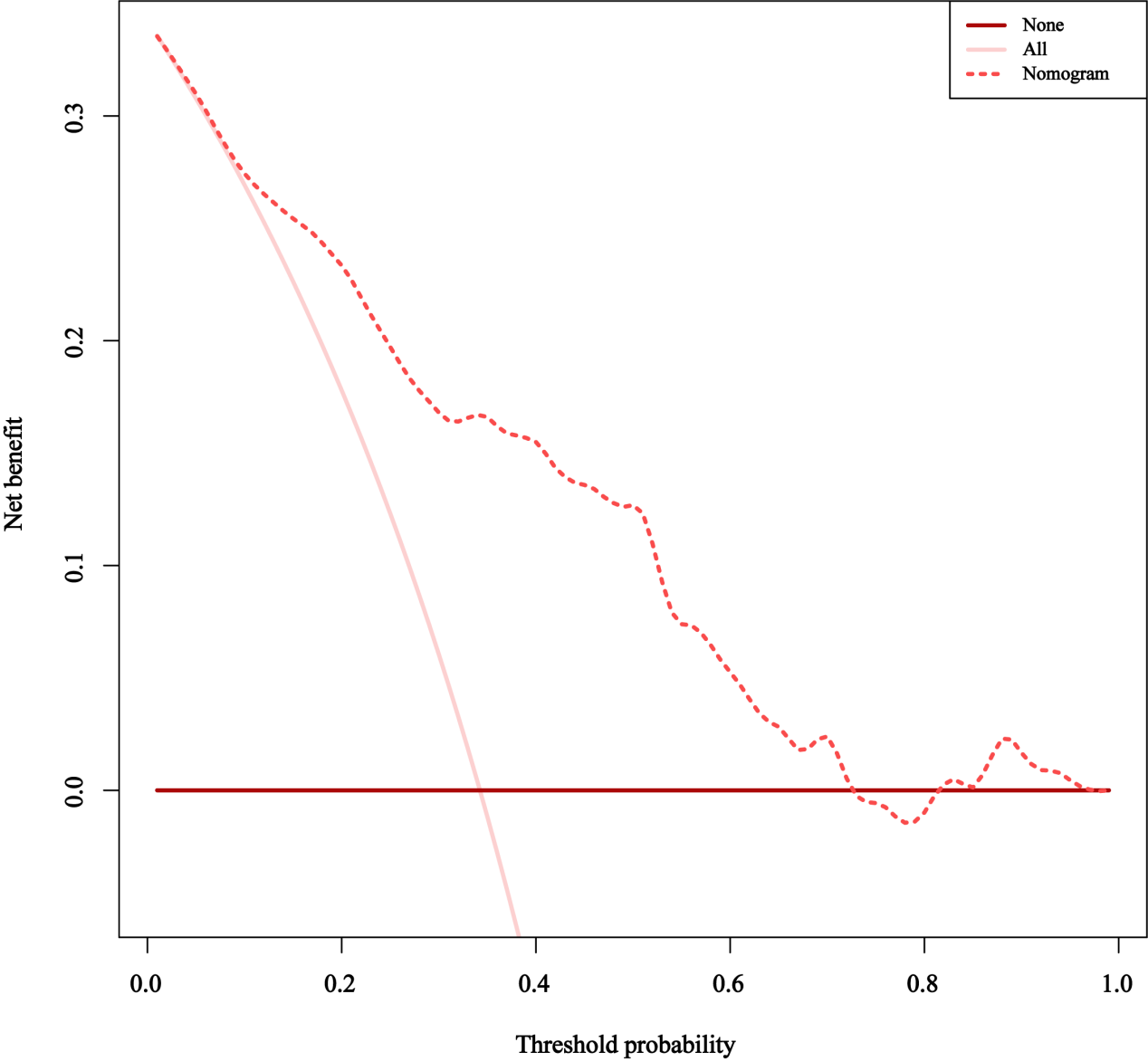
**

**Supplementary Fig. 3** The decision curve analysis of the nomogram for predicting major LARS.
